# Supplementary material for: Dynamic acoustic optimization of pulse tube refrigerators for rapid cooldown
Source: Nat Commun. 2024 Apr 23;15:3386. doi: 10.1038/s41467-024-47561-5 (PMC11039843; doi:10.1038/s41467-024-47561-5)
Supplement: Supplementary file 1 — Supplementary Information [file 41467_2024_47561_MOESM1_ESM.pdf]

Supplementary Information

Dynamic acoustic optimization of pulse tube refrigerators for rapid cooldown

Ryan Snodgrass, Vincent Kotsubo, Scott Backhaus, and Joel Ullom

National Institute of Standards and Technology, Boulder, CO 80305
Department of Physics, University of Colorado Boulder, Boulder, CO 80309

March 22, 2024

Supplementary Methods

A. Circuit analysis with square waves

Equations (1) and (2) were derived assuming sinusoidal waves with amplitude $p_{1,in}$ or $U_{1,in}$. Here we discuss the appropriate scaling of $\dot{E}_{2,n}$ when using those equations but for square waves of the same amplitude. A square wave $F(t)$ with amplitude 1 and angular frequency ω may be represented by an infinite series of sinusoidal waves:

$$F(t) = \frac{4}{\pi} \sum_{n=1,3,5...}^{\infty} \frac{1}{n} \sin(n\omega t). \tag{S1}$$

The components of the circuit shown in Fig. 2 are linear and follow superposition. Therefore, the acoustic power generated by each wave of the infinite series can be calculated individually using Eqs. (1) and (2), and the total acoustic power is the sum of the acoustic powers generated by each wave of the infinite series.

First consider the circuit when driven by a flow-rate source. The acoustic power generated by the wave at angular frequency ω is the same as given by Eq. (2) except multiplied by $(4/\pi)^2$ (from two $4/n\pi$ terms with $n = 1$). The acoustic power generated by the wave at angular frequency $n\omega$ is the same as Eq. (2) but multiplied by $(4/n\pi)^2$ and (accounting for the ω-dependent terms of Eq. (2)) $[(T_w/T_c+C_b/C_n)^2+(\omega RC_b)^2]/[(T_w/T_c+C_b/C_n)^2 + (n\omega RC_b)^2]$. If $\omega RC_b \gg T_w/T_c + C_b/C_n$ then the $n\omega$ component of the infinite series will not generate much acoustic power, as the above ratio is approximately $1/n^2$. In this scenario, the acoustic power at the network generated by all components of the infinite series could be estimated only by the ω component, resulting in $\dot{E}_{sq} \approx (4/\pi)^2 \dot{E}_{2,n}$.

Now consider the other extreme possibility, that $\omega RC_b \ll T_w/T_c + C_b/C_n$. In this case the power generated by the $n\omega$ wave is the same as Eq. (2) but multiplied by $(4/n\pi)^2$. This bounds the acoustic power generated by the square wave between two limits:

$$1.62 \approx (4/\pi)^2 < \frac{\dot{E}_{sq}}{\dot{E}_{2,n}} < 2, \tag{S2}$$

where 2 on the right side was substituted for $(4/\pi)^2 \sum_{n=1,3,5...}^{\infty} 1/n^2 = (4/\pi)^2\pi^2/8$. We used LTspice to simulate the circuit of Fig. 2 with both square and sinusoidal waves and confirmed that the above bounds are accurate.

Now we consider the circuit when driven by a pressure source. The acoustic power generated by the wave at angular frequency ω is the same as given by Eq. (1) except multiplied by $(4/\pi)^2$. The frequency-dependent coefficient $(\omega RC_n)^2/[1 + (\omega RC_n)^2]$ is approximately 1 when $(\omega RC_n)^2 \gg 1$. For such systems $\dot{E}_{sq}/\dot{E}_{2,n} \approx 2$ for the same reasons as discussed in the above paragraph. If $(\omega RC_n)^2 \ll 1$, then $\dot{E}_{sq}/\dot{E}_{2,n}$ can be significantly greater than 2. However, such a condition should be rare for actual PTRs because then little acoustic power is generated at the network: $\dot{E}_{2,n} \to 0$ as $(\omega RC_n)^2 \to 0$.

B. Simplified circuit analysis

Here we derive the acoustic power at the terminating network $\dot{E}_{2,n}$ of the simplified circuit shown in Fig. 2. From thermoacoustics [S1] we know

$$\dot{E}_{2,n} = \mathrm{Re}[\widetilde{p_{1,n}} U_{1,n}]/2 = \mathrm{Re}[p_{1,n}\widetilde{U_{1,n}}]/2, \tag{S3}$$

where $p_{1,n}$ is the pressure and $U_{1,n}$ is the volumetric flow rate at the inlet to the terminating network, and tildes represent the complex conjugate. Note that in this simplified circuit the entrance to the terminating network is at the same node as the regenerator's cold end.

The volumetric flow rate into the reservoir can be expressed by the pressure difference across the main impedance and also by the compliance of the terminating network's reservoir:

$$U_{1,n} = \frac{p_{1,n} - p_{1,r}}{R} = i\omega C_n p_{1,r}, \tag{S4}$$

where the main orifice impedance is R and is treated as purely real, $i = \sqrt{-1}$, C_n is the compliance of the reservoir, and $p_{1,r}$ is the pressure in the reservoir. The right hand side of Eq. (S4) is used to eliminate $p_{1,r}$, so that

$$U_{1,n} = p_{1,n} \frac{i\omega C_n}{1 + i\omega RC_n}. \tag{S5}$$

The above is inserted into Eq. (S3) and simplified to arrive at Eq. (1) from the main manuscript, which is the acoustic power at the entrance to the terminating network when the compressor and rotary valve system acts as a pressure source. The entrance of the terminating network is at the same pressure as the inlet, so $p_{1,n} = p_{1,in}$.

Now we consider the second case, when the compressor and rotary valve system acts as a flow-rate source. A useful preliminary result is

$$\dot{E}_{2,n} = \frac{R}{2}|U_{1,n}|^2, \tag{S6}$$

which is obtained by rearranging Eq. (S5) for $p_{1,n}$ and inserting into Eq. (S3). Continuity at the refrigerator inlet gives

$$U_{1,in} = i\omega C_b p_{1,n} + U_{1,n} T_w/T_c, \tag{S7}$$

where the flow rate at the warm end of the regenerator $U_{1,w}$ was substituted for $U_{1,c}T_w/T_c = U_{1,n}T_w/T_c$.

Equation (S5) is used to eliminate $p_{1,n}$ from Eq. (S7), and then it is possible to solve for $U_{1,n}$ in terms of the flow rate at the inlet to the refrigerator $U_{1,in}$:

$$U_{1,n} = \frac{U_{1,in}}{\frac{T_w}{T_c} + \frac{C_b}{C_n} + i\omega RC_b}, \tag{S8}$$

where C_b is the lumped compliance accounting for all compressible volume between rotary valve and terminating network. Inserting the above into Eq. (S6) and simplifying gives Eq. (2) from the main manuscript.

C. Acoustic power calculation

Five Endevco piezoresistive pressure transducers were installed in the ambient-temperature part of the pulse tube refrigerator; the locations of each may be seen in Fig. 1a. These sensors were calibrated in situ and read with an analog-to-digital converter at a sample rate of approximately 800 Hz.

Acoustic power in thermoacoustic devices is calculated [S1] as $\dot{E}_2 = \mathrm{Re}[p_1\widetilde{U_1}]/2$, where the pressure phasor is p_1, the volume flow rate phasor is U_1, and the tilde represents the complex conjugate. This expression is appropriate for sinusoidal waves with angular frequency ω.

The phasor describing the volume flow rate entering an RC network with neglible volume in the impedance is calculated [S1] as

$$U_1 = \frac{i\omega V}{\gamma p_m} p_{1,r}, \tag{S9}$$

where $i = \sqrt{-1}$ and $p_{1,r}$ is the pressure phasor in the reservoir volume V. When considering thermal-relaxation conductance near the surface of the volume, the above is modified [S2] as

$$U_1 = \frac{\omega}{\gamma p_m}\left[iV + (i+1)\frac{\gamma-1}{1+\epsilon_s}\frac{S\delta_\kappa}{2}\right]p_{1,r}, \tag{S10}$$

where S is the surface area, ϵ_s is a function of the thermal properties of fluid and solid, and δ_κ is the thermal penetration depth. In this work, $\epsilon_s \approx 0$ because the thermal conductivity and volumetric heat capacity of the reservoir material (aluminum) is much greater than helium's at 295 K and 1.4 MPa.

We are interested in signals that are not sinusoidal, so we proceed by switching to the time t domain. The equivalent of Eq. (S10) in the time domain is

$$U(t) = \frac{1}{\gamma\overline{p_r(t)}}\left[(V+\alpha)\frac{dp_r(t)}{dt} + \omega\alpha[p_r(t) - \overline{p_r(t)}]\right], \tag{S11}$$

where $\alpha = S\delta_\kappa(\gamma-1)/2(1+\epsilon_s)$, $p_r(t)$ is the measured pressure in the reservoir, and the overline represents the cycle mean. Equation (S11) says that the volume flow rate is split between two processes. The first $dp_r(t)/dt$ term is analogous to flow into a compliance but with a coefficient that adds the adiabatic compressibility of the bulk reservoir with the compressibility of the boundary layer, which is between the adiabatic and isothermal compressibilities. The second $p_r(t) - \overline{p_r(t)}$ term represents thermal-relaxation conductance in the reservoir's boundary layer [S1].

The instantaneous acoustic power at the entrance to the RC network is the pressure oscillation at the junction $p_j(t) - \overline{p_j(t)}$ multiplied by $U(t)$, so the cycle-mean acoustic power is

$$\dot{E}_{2,n} = \overline{[p_j(t) - \overline{p_j(t)}]U(t)}. \tag{S12}$$

The junction is where the bypass, warm end of the buffer tube, and main needle valve meet, as shown in Fig. 1a.

Although the pressure and flow rate waveforms of the PTR studied here are not exactly sinusoidal (see Fig. S16, for example), an approximate value for the volume flow rate amplitude into each reservoir $|U_{1,n}|$ is useful for the discussions around Fig. 8 and Fig. S14. We calculated $|U_{1,n}|$ as half the difference between the maximum and minimum of $U(t)$.

D. Thermosiphons

A thermosiphon was designed using the guidelines of the ASME BPVC (American Society of Mechanical Engineers Boiler and Pressure Vessel Code) and filled with either ethane, nitrogen, or argon. Approximate geometry of the thermosiphon may be seen in Fig. 6. The 304 stainless steel tube that connects the condenser to the evaporator had an inner diameter of approximately 1.3 cm. The condenser and evaporator were made of 101 copper. The thermosiphons were vacuum brazed (alloys Palcusil 15 and Cusil) and were hydrostatically pressure tested to 1.3 times the design pressure. The pressure tests were successful and showed no signs of leakage or permanent deformation.

The thermosiphon design pressure was 150 bar. The fill pressure at 295 K was approximately 134 bar, 134 bar, and 37 bar for nitrogen, argon, and ethane, respectively. The canister used to fill the ethane thermosiphon contained both saturated vapor and liquid at 295 K, so we used a scale (0.1 g precision) to track how much ethane was transferred from the canister, trying to condense only a minimal amount of ethane in the thermosiphon. We did not study how the fill ratio affected the performance of the thermosiphon. Each thermosiphon was connected to a gas manifold at room temperature through a small stainless steel tube, enabling measurement of the vapor pressure during cooldown.

We calculate the steady heat leak of each thermosiphon as approximately 115 mW when the condenser's temperature is 44 K and the evaporator's temperature is 4 K. This assumes the only mode of heat transfer is conduction through the stainless steel tube. As shown in Fig. 6, the length of the tube is much less than the distance between heat exchangers. It would be easy to decrease this heat leak if the thermosiphon was incorporated into the PTR manufacturing and the stainless steel tube was the same length as the second-stage regenerator.

After the cooling power measurements (Figs. 4 and 5) were completed but before the cooldown speed measurements (Fig. 7), two thermosiphons were installed between the first-stage heat exchanger and the copper load bolted to the second stage (Fig. 6). To minimize the thermal resistance between surfaces, the flanges of the thermosiphon were gold plated and a layer of indium foil (0.2 mm thick) was tightly pressed between each flange and its mating copper surface.

The performance of the thermosiphons (Fig. S8) was measured by comparing regulated cooling powers at the first and second stages when the thermosiphons were filled with a variety of fluids to when they were installed in the cryostat but evacuated. Figure S8 contains results for nitrogen, ethane, and argon-filled thermosiphons. Compared to nitrogen, argon did not improve the overall cooldown speed, so this fluid was not used in any results presented in the main manuscript. Nitrogen could be replaced with oxygen to achieve better temperature coverage

[S3] below the triple point of ethane, but we avoided this fluid for safety concerns. In applications where it is desirable to use only nonflammable fluids, trifluoromethane (R-23) may be used instead of ethane [S4]. We charged the ethane thermosiphon with 8.2 g of fluid—not significantly more than the amount of butane contained in a disposable cigarette lighter (about 5 g).

Figure 6 shows that the thermisiphons were rigidly connected between the first and second heat exchangers. We used COMSOL Multiphysics to check that differences in thermal contraction during cooldown would not cause any portion of the PTR or thermosiphons to stress beyond each material's yield strength.

E. Enthalpy removed and virtual copper mass

In addition to many copper masses, there was a significant amount of aluminum mounted on the first-stage heat exchanger, which is why it was not possible to simply compare the mass of copper mounted to each stage. The enthalpy removed from the first and second-stage loads during cooldown Δh was estimated using

$$\Delta h = \sum_i m_i \int_{4\,\mathrm{K}}^{295\,\mathrm{K}} c_i(T) dT, \qquad \text{(S13)}$$

where m_i is the mass of each solid and $c_i(T)$ is the specific heat capacity as a function of temperature. The enthalpy removed from the nitrogen and ethane in the thermosiphons was not considered in Δh because it was negligible compared to the enthalpy removed from the solids. For example, even with no virtual mass Δh_2 was about 1,500 kJ, while the enthalpy removed from the 8.2 g of ethane and 8.1 g of nitrogen in the thermosiphons was less than 10 kJ each (calculated using CoolProp [S5]).

To emulate copper masses on the second stage larger than the actual mass, heat was applied according to the change in enthalpy:

$$\dot{Q}_v = -m_v c_c(T_{i-1}) \frac{T_i - T_{i-1}}{t_i - t_{i-1}}, \qquad \text{(S14)}$$

where m_v is the virtual mass, c_c is the specific heat capacity of copper [S6], T_i is the current temperature of the copper load, and $t_i - t_{i-1}$ is the time difference between measurements. The heat $\dot{Q}_v$ was updated every 2 s and applied to the actual copper load using an array of resistive heaters.

Supplementary Figures

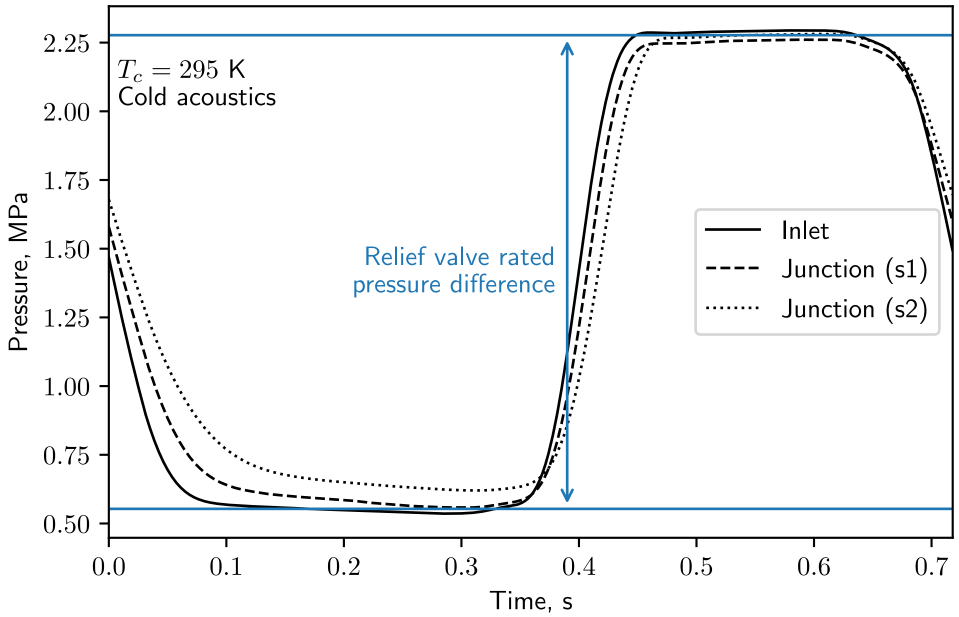

Figure S1: Pressure oscillation at the inlet and junctions. Pressure at the inlet to the PTR (solid line), first-stage junction (dashed line), and second-stage junction (dotted line) over one cycle. A junction is where the bypass, warm end of the buffer tube, and inlet to the *RC* network meet.

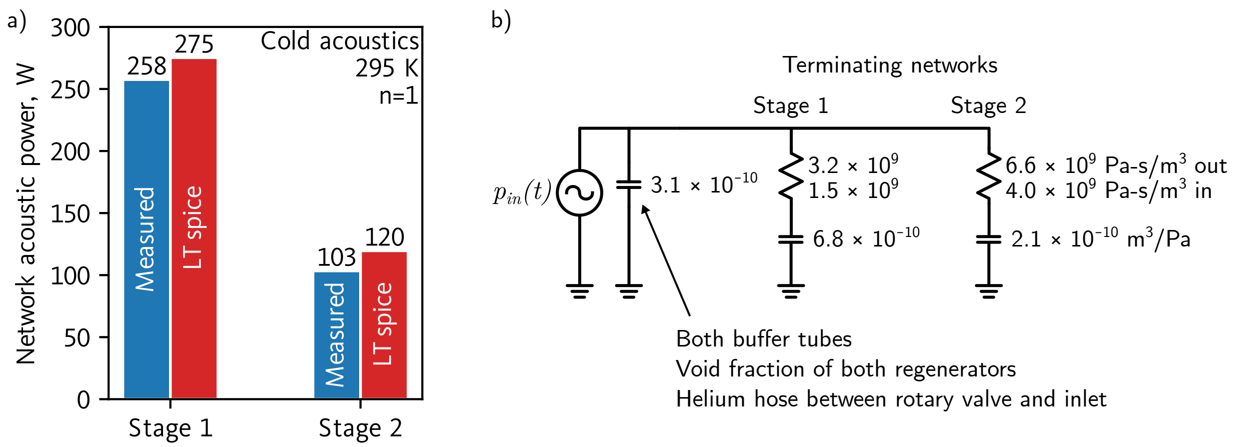

Figure S2: Comparison to LTspice and estimates for acoustic parameters. a) Comparison of the measured network acoustic power with that calculated using software LTspice. The simulated LTspice circuit b) is similar to Fig. 2 except both terminating networks are modeled. Because the input waveform is pressure limited at 295 K and with cold acoustics, it is not necessary to include attenuation of U_1 through the regenerator, since it does not affect $\dot{E}_{2,n}$ (Eq. (1)). The pressure at the inlet $p_{in}(t)$ was measured in the commercial PTR and input directly into LTspice. The impedances of the main needle valves were measured (Fig. S5) and are asymmetric depending on the direction of flow—the schematic shows the values used depending on whether flow was leaving or entering the terminating networks. The compliances were calculated assuming a mean pressure p_m of 1.4 MPa and using the measured geometry of the reservoirs, regenerators, buffer tubes, and helium hose between rotary valve and PTR inlet. Regenerator volumes were assumed to be isothermal with compressibility $1/p_m$. Buffer tube volumes were assumed adiabatic with compressibility $1/\gamma p_m$. The compliance of the reservoirs was calculated assuming an adiabatic bulk and a boundary layer with compressibility equal to the mean of the adiabatic and isothermal compressibilities.

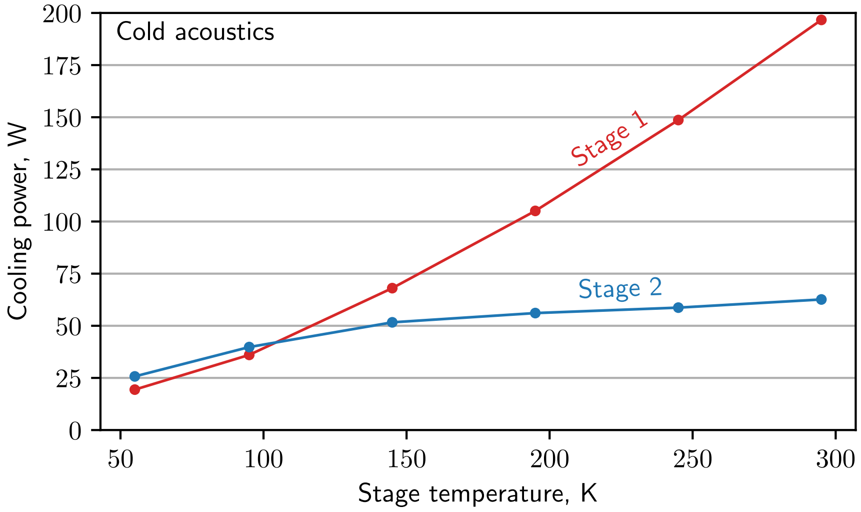

Figure S3: Cooling power with cold acoustics. Cooling power at the first and second stages when regulated to the same temperature. Acoustic parameters were set to optimize performance at base temperature.

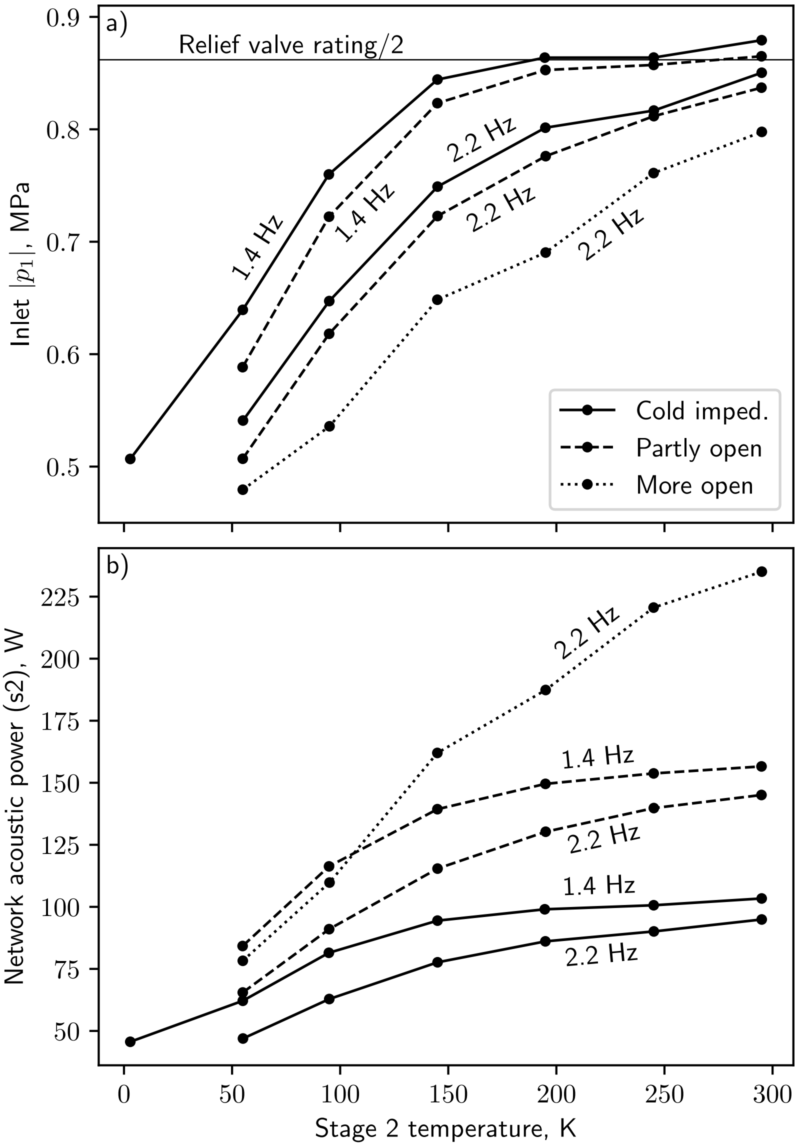

Figure S4: Pressure and acoustic power measurements for a variety of acoustic settings. a) The pressure amplitude measured at the inlet to the PTR as a function of the second-stage temperature and under a variety of acoustic settings. The solid line was collected with the main needle valve impedances set to produce the best low-temperature performance (Fig. S6), while dashed and dotted lines are for progressively opened needle valves (smaller R). b) The acoustic power measured at the inlet to the second-stage terminating network ($\dot{E}_{2,n2}$).

Regarding agreement with Eq. (1) (Fig. 2 driven by a pressure source): At 1.4 Hz and between 295 K and 195 K, $|p_1|$ at the PTR's inlet is nearly fixed at half the relief valve's rated pressure. Under these conditions, $\dot{E}_{2,n2}$ changes little with temperature, which matches the expectation from Eq. (1). With cold impedance, $\dot{E}_{2,n2}$ at 295 K is only 103 W, but when R is decreased (partly open) $\dot{E}_{2,n2}$ increases to 157 W, which also matches the (qualitative) expected change. At 1.4 Hz and below 195 K, $|p_1|$ drops and the compressor no longer functions as a pressure source.

Regarding agreement with Eq. (2) (Fig. 2 driven by a flow-rate source): First consider the data at 95 K and fixed R (compare the two dashed lines). An increase in frequency here causes $\dot{E}_{2,n2}$ to drop, suggesting that the $\omega R C_b$ term in the denominator of Eq. (2) is significant. At this operating point and considering only the second-stage network, $\omega R C_b$ is estimated to be 8.1 while $T_w/T_c + C_b/C_n$ is estimated to be 4.6 (Figs. S2 and S5). Higher frequency is disadvantageous because the volume between rotary valve and terminating network must be compressed more often, so less of the flow entering the PTR generates $\dot{E}_{2,n}$ [S7]. All $\dot{E}_{2,n2}$ curves decrease as the cold-end temperature does—but especially when $|p_1|$ at the inlet is not limited by the relief valve. This arises from the attenuation of flow in the regenerators and the corresponding growth of the T_w/T_c term in the denominator of Eq. (2). The T_w/T_c term is most significant when $\omega R C_b$ is smallest, which matches the measured trend for $\dot{E}_{2,n2}$: the dotted line (with smallest R) is most sensitive to temperature changes.

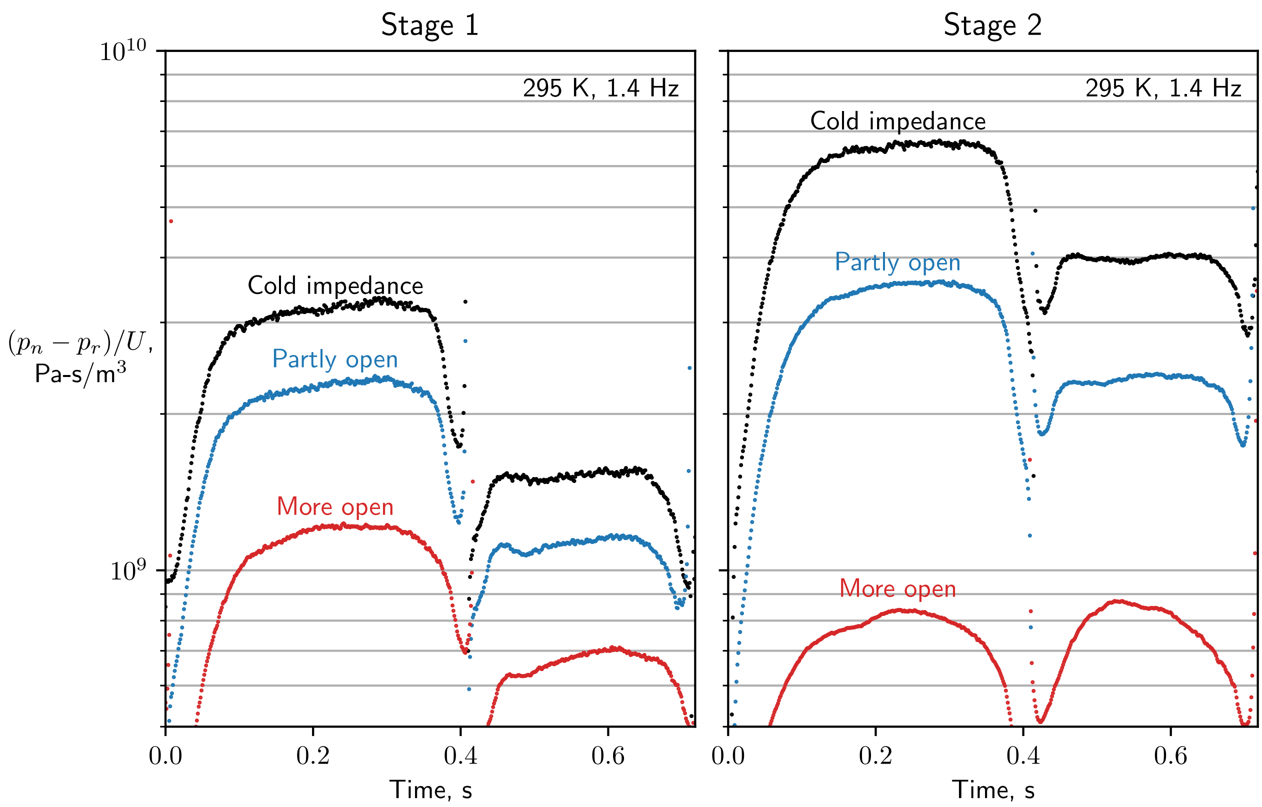

Figure S5: Estimates for the impedances of the main needle valves. The pressure difference across the main needle valves divided by the calculated volume flow rate, i.e., estimates for acoustic impedance. The pressure difference was directly measured and the flow rate was calculated using Eq. (S11). During the first part of the cycle flow is leaving the terminating network and during the second part flow is entering the terminating network.

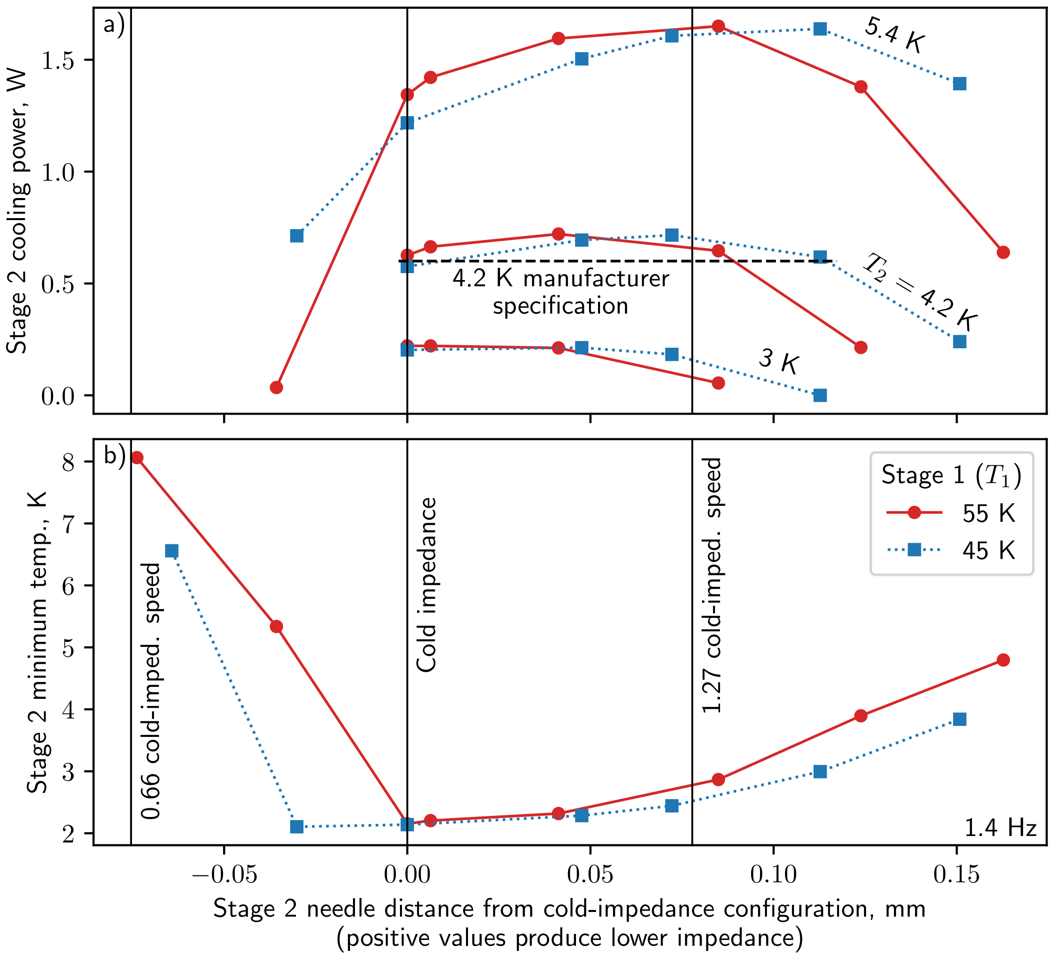

Figure S6: Determination of optimal acoustic settings at base temperature. The cooling power a) and temperature of the second stage with no heat applied b) as functions of that stage's main-orifice needle position. These measurements were completed with the first stage regulated to 55 K (red circles) and 45 K (blue squares), and f fixed at 1.4 Hz. Cooling power was measured for three different cold-end temperatures: 5.4 K, 4.2 K, and 3 K. The dashed horizontal line in a) shows the cooling power guaranteed by the manufacturer (0.6 W) when the first stage is regulated to 55 K and the second 4.2 K. The vertical lines show the needle position for the cold-impedance configuration and two other needle positions where the cooldown speed was measured. The right vertical line shows a needle position that resulted in a cooldown speed 1.27 times cold impedance, while the left vertical line shows a position resulting in 0.66 times the cold-impedance speed. These results show that the default cooldown speed is a strong function of the optimization performed by the manufacturer. Additionally, optimization is dependent on the first and second-stage temperatures.

To choose the needle positions for the cold-impedance configuration we first found the second-stage bypass and first-stage main impedances that produced the lowest combination of first and second-stage temperatures, with greater importance given to the second-stage temperature. We then used the experiments shown above to find the second-stage main needle position that resulted in the lowest second-stage temperature while still maintaining the manufacturer-guaranteed cooling at 4.2 K.

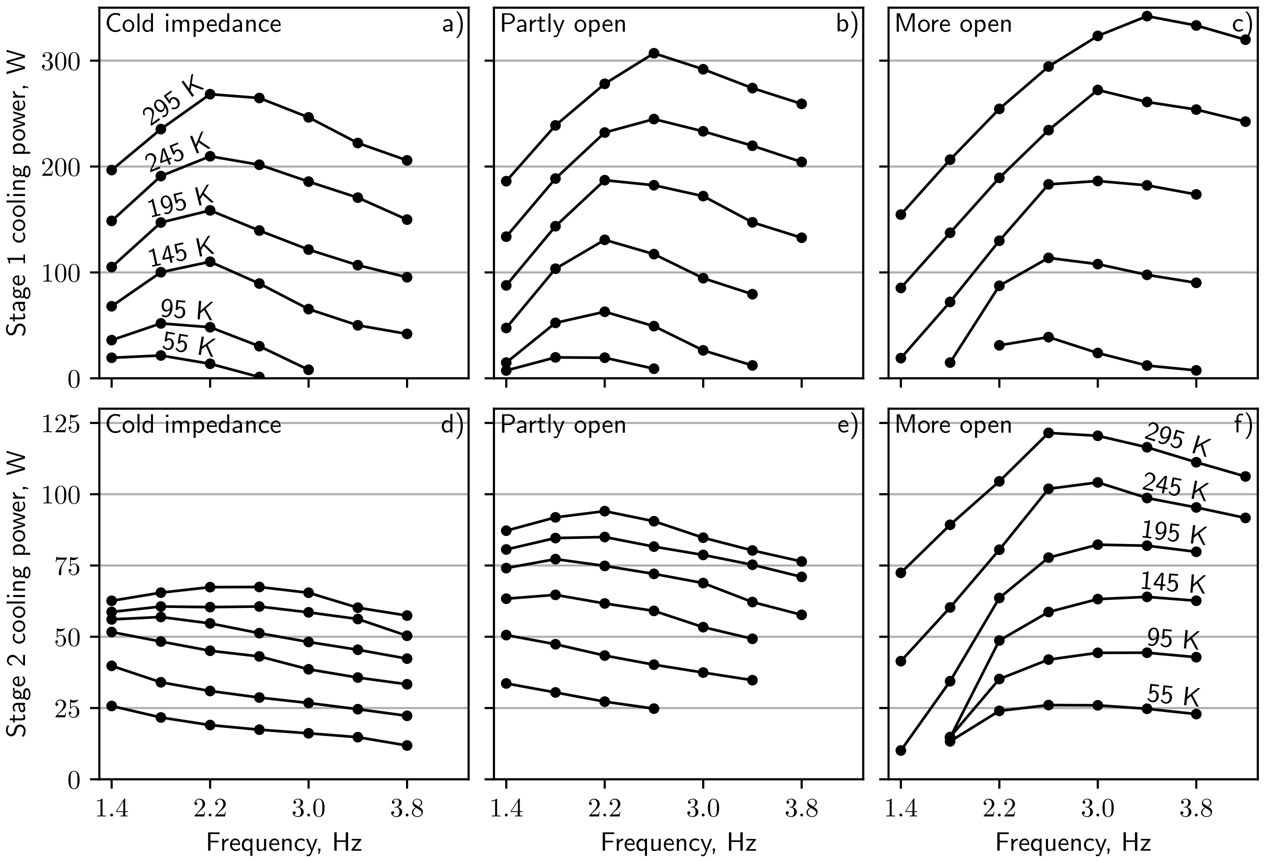

Figure S7: Cooling power for different acoustic settings. Cooling power at the first stage (top row) and second stage (bottom row) as a function of frequency. The left column shows when the main impedances were set to give the best performance at low temperature, while the middle and right columns are for progressively smaller main impedances (see main text for exact positions). The top line in each subplot shows the cooling power at 295 K. For subplot c) there are only five curves because the first stage could not cool to 55 K even with no heat applied.

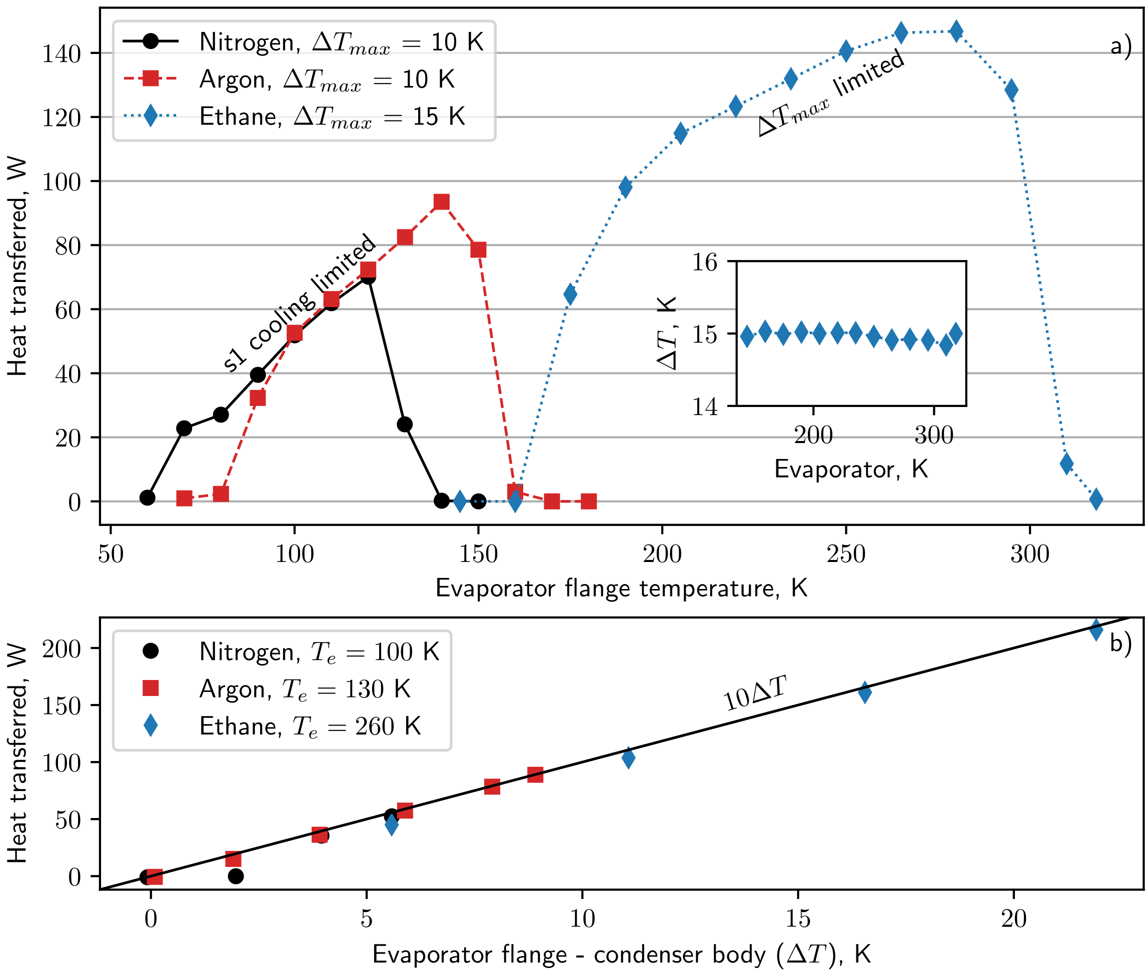

Figure S8: Thermosiphon performance (temperature regulated). Measured performance of a thermosiphon when filled with nitrogen, argon, or ethane. To measure the heat transferred by the thermosiphon we compared cooling power measurements at the first and second stage when the thermosiphons were filled with these fluids to when the thermosiphons were under vacuum. When filled with fluids, the cooling power increase at the second stage was very close to the cooling power decrease at the first stage. Subplot a) gives the heat transferred between evaporator and condenser as a function of evaporator temperature. For nitrogen and ethane, we limited the temperature difference between evaporator and condenser ΔT to 10 K, while for ethane we limited ΔT to 15 K. The heat transferred by the ethane thermosiphon was limited by this temperature difference (as shown in the inset), while nitrogen and argon transferred more heat than the first stage could provide. Subplot b) plots the heat transferred as a function of ΔT when the evaporator temperature T_e was regulated to a single value and the condenser temperature was regulated to a variety of values. All three fluids achieve nearly 10 W/K when T_e was not too close to the triple or critical temperatures, as shown by the $10\Delta T$ line.

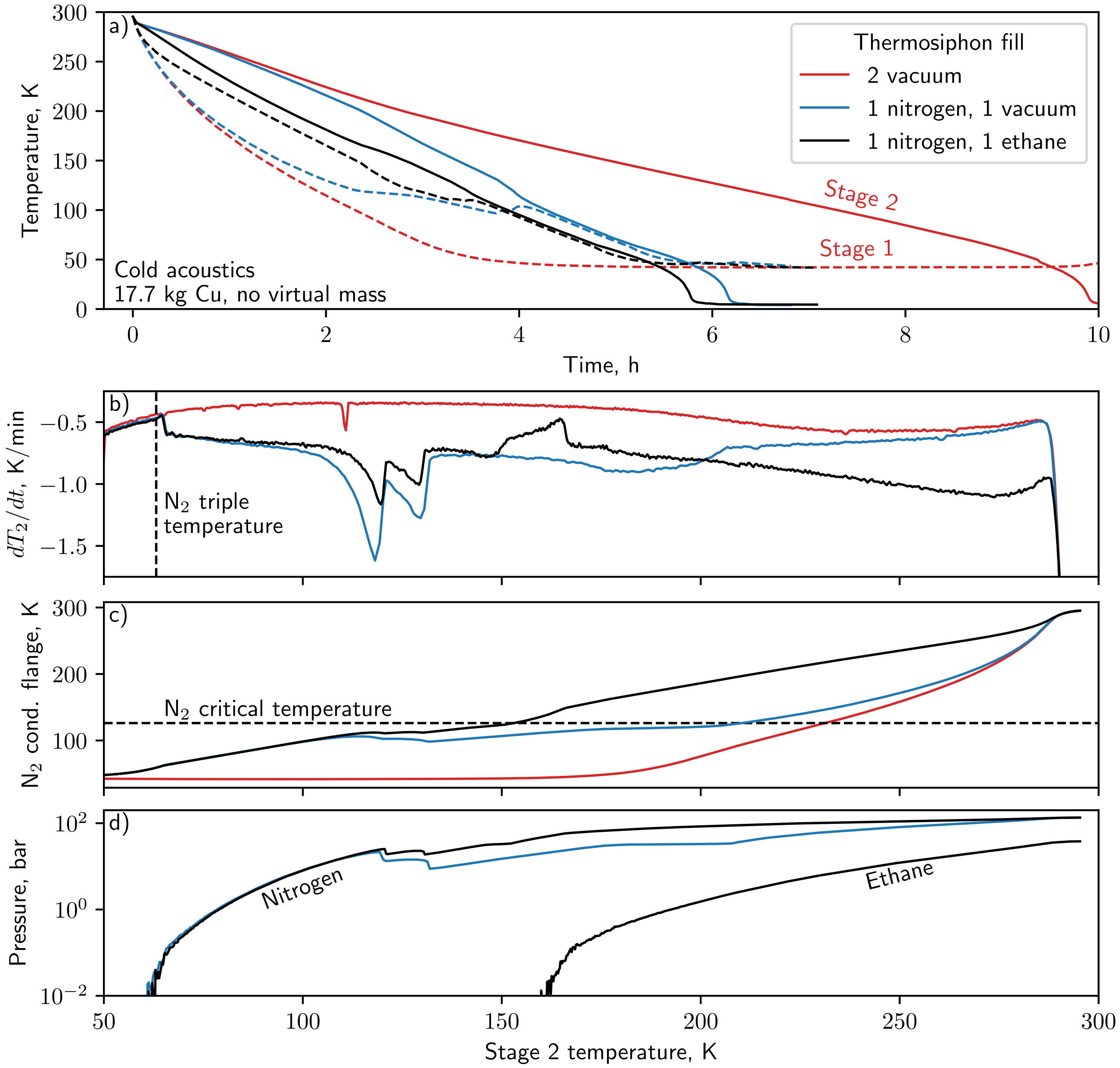

Figure S9: Thermosiphon performance (cooldowns). Three cooldowns with identical cryostats and (fixed) acoustic parameters except with the two thermosiphons filled with different fluids. Subplot a) shows the second-stage temperature T_2 (solid lines) and first-stage temperature T_1 (dashed lines) as a function of time. Subplots b) through d) are functions of T_2 and show the second-stage cooling rate, the temperature of the nitrogen thermosiphon's condenser, and pressure in each thermosiphon, respectively. Red lines are for both thermosiphons under vacuum and serve as a control with minimal heat transfer between stages. The blue lines show when one of the thermosiphons is filled with nitrogen, so the deviation of the blue lines from the red lines is explained by heat transferred through the nitrogen thermosiphon. Although nitrogen does not condense at temperatures above 126 K, heat transfer appears to start when $T_1 < 200$ K and $T_2 < 270$ K. This observation is possibly explained by Rayleigh-Benard convection, as the nitrogen in the (colder) condenser is denser and above that in the evaporator. The kink of the blue curve in b) near $T_2 = 210$ K corresponds to the first condensation of nitrogen—c) shows that the condenser is near 126 K at this time—greatly increasing the cooldown rate of the second stage. When the other thermosiphon was filled with ethane (black lines), the second stage cooled more quickly before the condensation of nitrogen but more slowly afterwards (compared to the blue lines). The latter effect occurs because the temperature difference between stages is greater when nitrogen condensation starts if the second thermosiphon is under vacuum than if it is filled with ethane. Near the triple temperature of nitrogen the three dT_2/dt curves collapse onto each other, indicating minimal heat transfer through the thermosiphons.

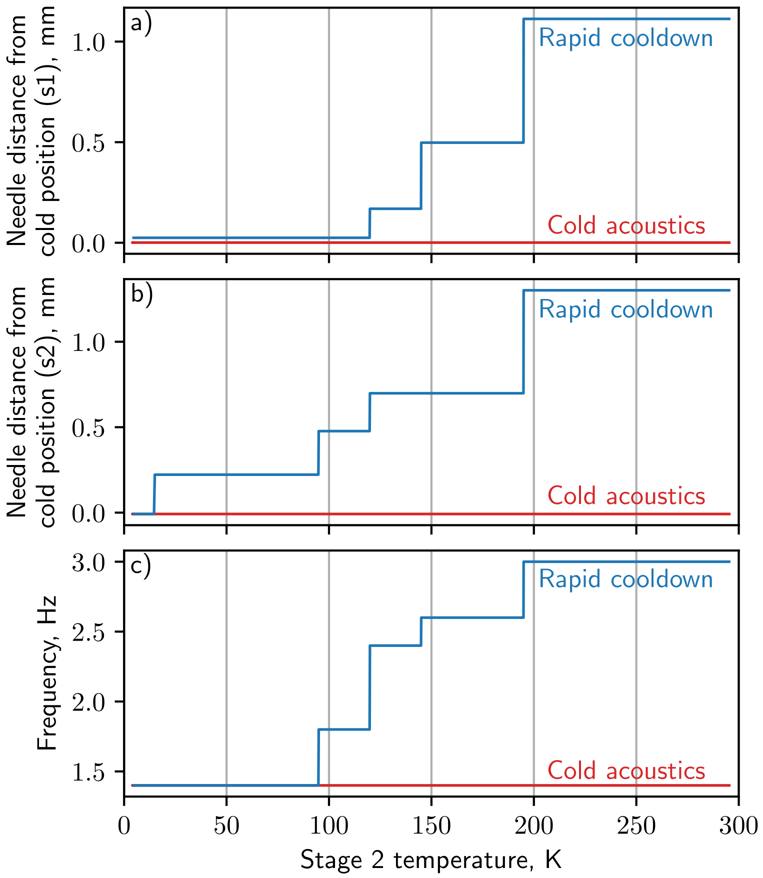

Figure S10: Cooldown procedure. Dynamic acoustic procedure for the rapid cooldowns shown in Fig. 7 and Fig. S12. An automated algorithm changed the position of the main-orifice needles of the first a) and second b) stages as a function of the second-stage temperature. Positive needle positions correspond to smaller R. The frequency of the rotary valve was also changed c).

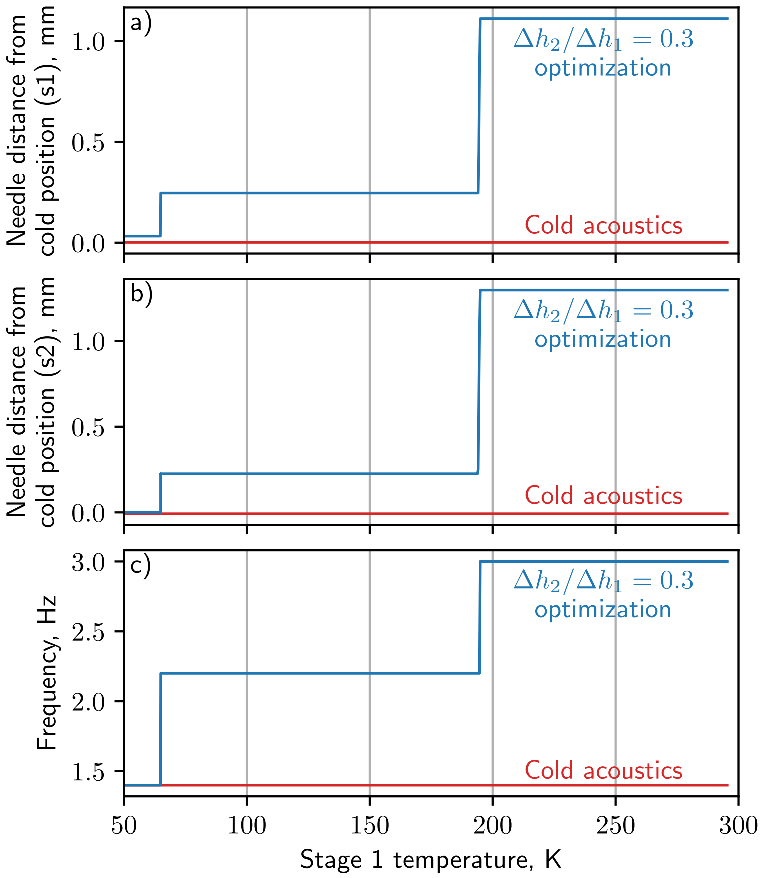

Figure S11: Cooldown procedure (small second-stage load). Dynamic acoustic procedure for the $\Delta h_2/\Delta h_1 = 0.3$ datapoint in Fig. 7b and Fig. S12. An automated algorithm changed the position of the main-orifice needles of the first a) and second b) stages as a function of the first-stage temperature. Positive needle positions correspond to smaller R. The frequency of the rotary valve was also changed c).

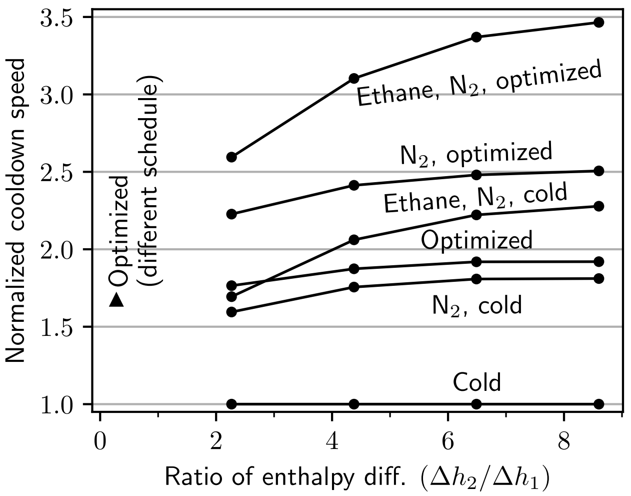

Figure S12: Overall cooldown speeds (all experiments). Cooldown speed normalized by the speed with cold acoustics and no heat transfer between stages. Lines are labeled with each thermosiphon's fluid (one fluid per thermosiphon and vacuum if no fluid specified) followed by the acoustic setting: either cold acoustics or optimized acoustics (Fig. S10). For the $\Delta h_2/\Delta h_1 = 0.3$ data point, the needle and frequency schedule was unique (Fig. S11).

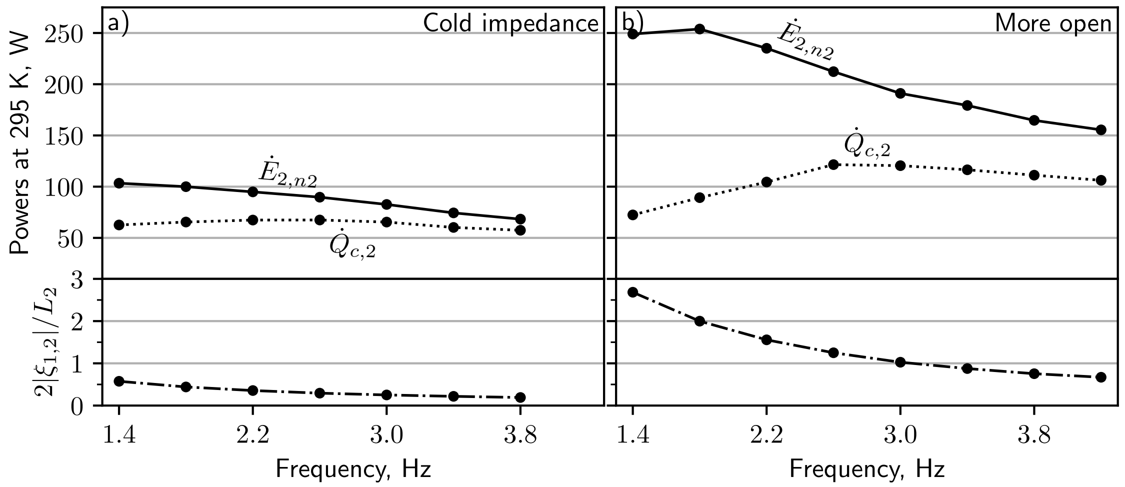

Figure S13: Cooling power loss at the second stage. Second-stage cooling power and acoustic power at the terminating network inlet as a function of f. Subplot a) is for cold impedance and b) is for decreased impedance (more open). The bottom row shows the approximate stroke of the helium in the second-stage buffer tube normalized by the buffer tube length.

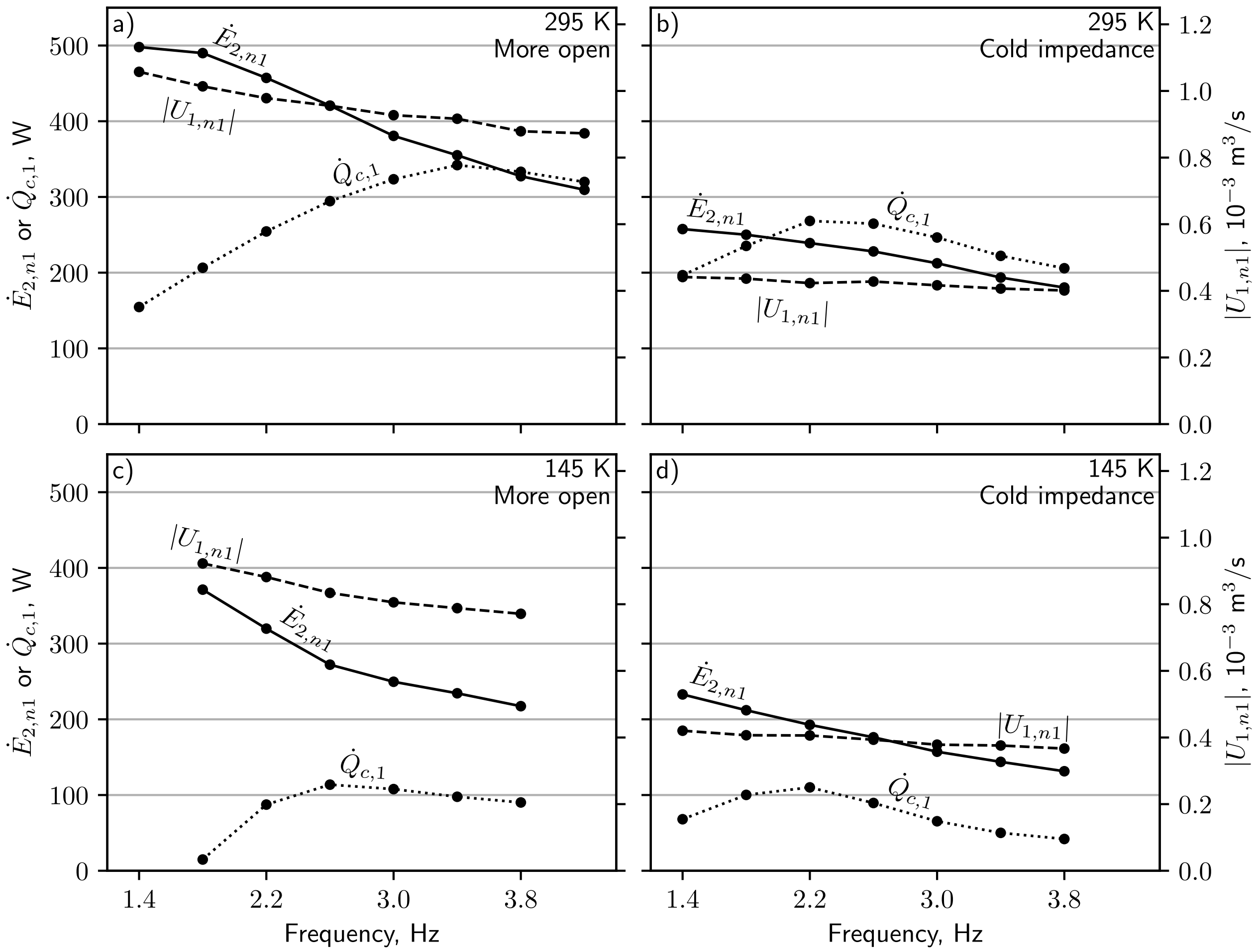

Figure S14: Estimates of network acoustic power and flow-rate amplitude for a variety of acoustic settings. Acoustic power at the first-stage network $\dot{E}_{2,n1}$, cooling power at the first stage $\dot{Q}_{c,1}$, and amplitude of the volume flow rate at the first-stage network $|U_{1,n1}|$ (Eq. (S11)) as a function of frequency. The top row plots these quantities at 295 K while the bottom row does so at 145 K. The right column was collected with the needles set to their cold-impedance positions while the left column shows results with more-open needle valves.

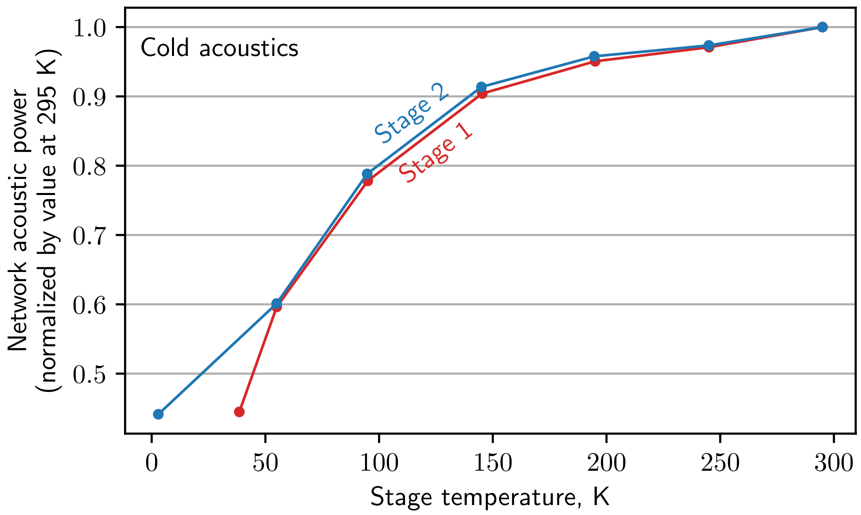

Figure S15: Acoustic power change with temperature. Normalized acoustic power at the inlet to the *RC* network of each stage, as a function of temperature. Acoustic parameters were set to optimize performance at base temperature.

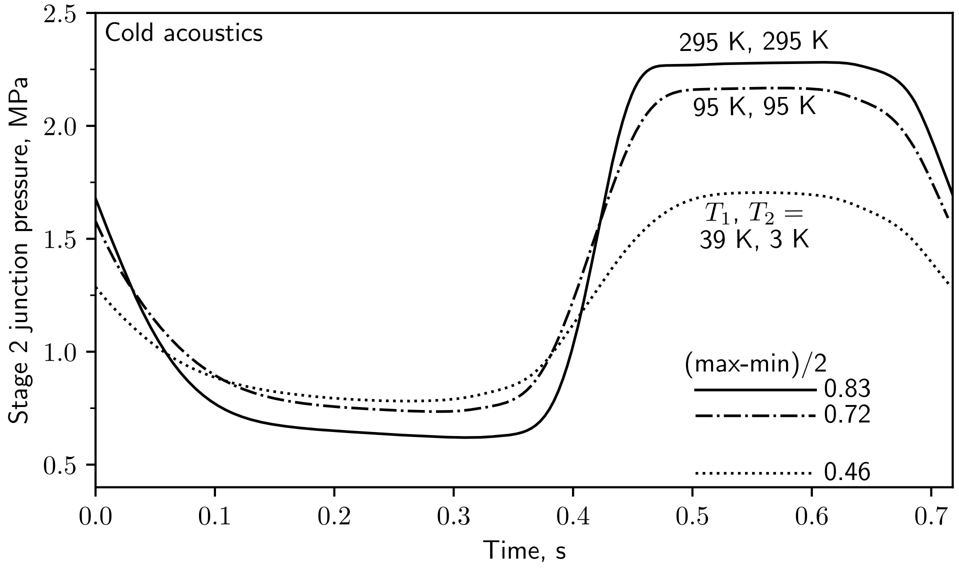

Figure S16: Change in pressure amplitude with temperature. Pressure measured at the second-stage junction for a variety of temperatures. The first stage and second-stage temperatures are T_1 and T_2, respectively. A junction is where the bypass, warm end of the buffer tube, and inlet to the RC network meet. Horizontal lines on the bottom right show the approximate pressure amplitude.

Supplementary References

[S1] G. W. Swift, *Thermoacoustics: A Unifying Perspective for Some Engines and Refrigerators*, 2nd ed. (Springer International Publishing, 2017).

[S2] B. Ward, J. Clark, and G. Swift, *Users Guide for DeltaEC: Design Environment for Low-amplitude Thermoacoustic Energy Conversion*, (2017) www.lanl.gov/thermoacoustics.

[S3] F. C. Prenger et al., "Heat Pipes for Enhanced Cooldown of Cryogenic Systems", in *Cryocoolers 9*, edited by R. G. Ross (Springer US, Boston, MA, 1997), pp. 831–839.

[S4] J. Lee, Y. Kim, and S. Jeong, "Transient thermodynamic behavior of cryogenic mixed fluid thermosiphon and its cool-down time estimation", Cryogenics **50**, 352–358 (2010).

[S5] I. H. Bell et al., "Pure and Pseudo-pure Fluid Thermophysical Property Evaluation and the Open-Source Thermophysical Property Library CoolProp", Industrial & Engineering Chemistry Research **53**, 2498–2508 (2014).

[S6] N. J. Simon, E. S. Drexler, and R. P. Reed, *Properties of copper and copper alloys at cryogenic temperatures. Final report*, PB-92-172766/XAB; NIST/MONO-177 (Feb. 1, 1992).

[S7] V. Kotsubo, "Real and Reactive Flows in Regenerative Cryocoolers", Cryocoolers 21, 245–253 (2021).
